# A nationwide survey of hereditary angioedema due to C1 inhibitor deficiency in Italy

**DOI:** 10.1186/1939-4551-8-S1-A184

**Published:** 2015-04-08

**Authors:** Andrea Zanichelli, Francesco Arcoleo, Maria Pina Barca, Paolo Borrelli, Maria Bova, Mauro Cancian, Marco Cicardi, Enrico Cillari, Caterina De Carolis, Tiziana De Pasquale, Isabella Del Corso, Ilaria Massardo, Paola Minale, Vincenzo Montinaro, Sergio Neri, Roberto Perricone, Stefano Pucci, Paolina Quattrocchi, Oliviero Rossi, Massimo Triggiani

**Affiliations:** 1 Università Di Milano, Ospedale Luigi Sacco, Italy; 2 Ospedali Riuniti Villa Sofia-Cervello, Italy; 3 AUO Cagliari, Italy; 4 Ospedale U. Parini, Aosta, Italy; 5 A.O.U. Federico II, Napoli, Italy; 6 Università Degli Studi Di Padova, Italy Azienda Ospedaliera San Giovanni-Addolorata Roma, Italy; 7 Presidio Ospedaliero Di Civitanova Marche, Italy; 8 Azienda Ospedaliera Universitaria Pisana (AOUP), Italy; 9 Università Degli Studi Di Firenze, Italy; 10 Irccs San Martino, Genova, Italy; 11 Azienda Ospedaliero-Universitaria "Consorziale Policlinico" Bari, Italy; 12 Policlinico Universitario Di Catania, Italy; 13 Policlinico Tor Vergata, Roma, Italy; 14 Policlinico Universitario, Messina, Italy; 15 Aou Careggi, Firenze, Italy; 16 Universita Degli Studi Di Napoli, Italy

## Background

Hereditary angioedema due to C1-inhibitor deficiency (C1-INH-HAE type I) or dysfunction (C1-INH-HAE type II) is a rare disease characterized by recurrent episodes of edema with an estimated frequency of 1:50,000 in the global population without racial or gender differences. In this study we present the results of a nationwide survey of C1-INH-HAE patients referring to 17 Italian centers, the Italian network for C1-INH-HAE, ITACA

## Methods

Italian patients diagnosed with C1-INH-HAE from 1973 to 2013 were included in the study. Diagnosis of C1-INH-HAE was based on family and/or personal history of recurrent angioedema without urticaria and on antigenic and/or functional C1-INH deficiency.

## Results

983 patients (53% female) from 376 unrelated families were included in this survey. Since 1973, 63 (6%) patients diagnosed with C1-INH-HAE died and data from 3 patients were missing when analysis was performed. Accordingly, the minimum prevalence of HAE in Italy in 2013 is 920:59,394,000 inhabitants, equivalent to 1:64,935. Compared to the general population, patients are less represented in the early and late decades of life: men start reducing after the 5^th^ decade and women after the 6^th^. Median age of patients is 45 (IQ 28-57), median age at diagnosis is 26 years (IQ 13-41). C1-INH-HAE type 1 are 87%, with median age at diagnosis of 25 (13-40); type 2 are 13% with median age at diagnosis of 31 (IQ 16-49). Functional C1INH is ≤50% in 99% of patients. Antigen C1INH is ≤50% in 99% of type 1. C4 is ≤50% in 96% of patients.

## Conclusions

This nationwide survey of C1-INH-HAE provides for Italy a prevalence of 1: 64,935. C1-INH-HAE patients listed in our database seem to have a shorter life expectancy than the general population. Since angioedema symptoms usually start during puberty, the estimated delay in diagnosis is higher than 10 years. The chance of having C1-INH-HAE with C4 plasma levels >50% is < 0.05.

